# Comparison of hypertonic saline versus normal saline on cytokine profile during CABG

**DOI:** 10.1186/2008-2231-20-49

**Published:** 2012-10-08

**Authors:** Mahnaz Mazandarani, Fardin Yousefshahi, Mohammad Abdollahi, Hadi Hamishehkar, Khosro Barkhordari, Mohammad Ali Boroomand, Arash Jalali, Arezoo Ahmadi, Reza Shariat Moharari, Mona Bashirzadeh, Mojtaba Mojtahedzadeh

**Affiliations:** 1 Department of Pharmacotherapy, Faculty of Pharmacy, Tehran University of Medical Sciences, Tehran, Iran; 2 Department of Anesthesia and critical care, Faculty of Medicine, Tehran University of Medical Sciences, Tehran, Iran; 3 Pharmaceutical Sciences Research Center, Tehran University of Medical Science, Tehran, Iran; 4 Department of Pharmacotherapy, Faculty of Pharmacy, Tabriz University of Medical Science, Tabriz, Iran; 5 Department of Pathology, Faculty of Medicine, Tehran University of Medical Science, Tehran, Iran; 6 Department of Epidemiology & Biostatistics, Faculty of Statistics, Tehran University of Medical Sciences, Tehran, Iran; 7 Department of Pharmacotherapy, School of Pharmacy, Islamic Azad University of Pharmaceutical Sciences, Tehran, Iran

**Keywords:** CABG, CPB, Hypertonic saline 5%, Inflammation, IL-6, IL-10

## Abstract

**Background and the purpose of the study:**

Blood contact with artificial surfaces of the extracorporeal circuit and ischemia-reperfusion injury in CABG with CPB, may lead to a systemic inflammatory response. Hypertonic saline have been recently investigated as a fluid in order to decrease inflammatory response and cytokines generation in patients undergo cardiac operations. Our purpose is to study the prophylactic effect of HS 5% infusion versus NS on serum IL-6 as an inflammatory & IL-10 as an anti-inflammatory biomarker in CABG patients.

**Methods:**

The present study is a randomized double-blinded clinical trial. 40 patients undergoing CABG were randomized to receive HS 5% or NS before operation. Blood samples were obtained after receiving HS or NS, just before operation, 24 and 48 hours post-operatively. Plasma levels of IL-6 and IL-10 were measured by ELISA.

**Results and major conclusion:**

Patients received HS had lower levels of IL-6 and higher level of IL-10 compared with NS group, however these differences were not statistically significant. Results of this study suggest that pre-treatment with small volume hypertonic saline 5% may have beneficial effects on inflammatory response following CABG operation.

## Introduction

Infusion of Hypertonic saline (HS) solution increases serum osmolarity and markedly intravascular and interstitial fluid volume expansion, which causes improving hemodynamic status [1]. Fluid resuscitation with various concentrations of HS solutions (1.8% - 7.5%) has been investigated in different types of hypovolemic shock [1]; pre-operative, intra-operative and post-operative fluid therapy [2], burn injury and also septic shock [1]. HS is inexpensive and has no risk of anaphylactoid reactions compared with other artificial plasma volume expanders. There is no risk of transmission of infectious agents compared with human plasma [3]. Rapid correction of intravascular volume is achieved with a small infused volume (4 ml/kg) [1].

Recent studies demonstrated immunomodulatory effects of hypertonic saline by blunting neutrophil activation and reducing cytokine production [4,5].

Cardiac surgery with cardiopulmonary bypass (CPB) leads to acute changes in the composition and volume of body fluid compartments. CPB dilutes serum proteins, decreases the plasma colloid osmotic pressure and reduces endothelial integrity [6]. This causes fluid shifts from the intravascular to extravascular space and leads to a 33% increase in extravascular fluid space and tissue edema [7]. Complement activation following with systemic inflammatory response syndrome (SIRS) occurs during extracorporeal circulation [6]. Depending on the severity, inflammatory response can cause cerebral, myocardial, pulmonary and renal dysfunction [6].

In this study it was hypothesized that administration of HS just before coronary artery bypass surgery (CABG) was be helpful in decreasing of generated inflammatory cytokines because of increasing in intravascular volume and tissue perfusion.

## Material & methods

*Study design & Patient population:* The present study is a randomized double-blinded clinical trial. 40 patients <70 years old, undergoing elective CABG surgery admitted in Tehran Heart Center, Tehran University of Medical Sciences, between December 2010 & March 2011, entered into the study. Patients were randomly assigned to control (A) and intervention (B) groups, based on a computerized randomization sequence form. All patients had undergo necessary clinical and paraclinical examinations including: CBC, Diff, Electrolytes, PFT, LFT, renal function tests, chest X ray, ECG, Cardiac Enzyme Assay, PT, PTT, INR, thyroid tests, blood group and matching, Carotid Doppler,…, patient educations, consultations, and preparing processes before surgery.

All the perioperative cares and also surgeries were carried out by the same (single) surgeon and intensivist who were blinded to the treatment groups. Patients also were blinded to the choice of fluids.

All patients had ejection fraction (EF) >35% and their serum creatinine were between 0.5-1.5 mg/dl pre-operatively. Our research was approved by Pharmaceutical Sciences Research Center ethics committee by ethical code of 89-7-7:15–1 according to the declaration of Helsinki and written informed consent was obtained from the legal guardian of each patient before enrollment. Exclusion criteria included any concomitant operation, recent myocardial infarction (during 6 months), emergent surgery, urgent or elective intubation before surgery, unstable hemodynamic status, recent cerebral vascular accident, neurological complications or consciousness disorder or significant neuralgic defect, head trauma, uncontrolled diabetes mellitus, congenital heart disorder, blood transfusion before operation, pre-operative infection, serum creatinine >1.5 mg/dl or GFR < 50, Na > 150 mmole/dl, BMI > 40 and Chronic Obstructive Pulmonary Disease (COPD) or abnormal pulmonary function test [respiratory distress, Carbon Dioxide Pressure (PCO_2_) > 45 mmHg and Oxygen Pressure (PO_2_) < 60 mmHg, Forced Expiratory Volume in 1 second (FEV_1_) < 60% or Forced Vital Capacity (FEV_1_/ FVC) < 60% and Vital Capacity (VC) <50%].

Patients were randomized to receive 100 ml hypertonic saline (5%) + 400 ml normal saline (0.9%) equivalent to 155 mmole NaCl (group B) or 500 ml normal saline (0.9%) equivalent to 77 mmole NaCl (group A); in a uniform packages from a peripheral venous line in upper arm during 6 hour before surgery.

All preparation and treatment measurements were performed in a unique manner based on the attending hospital protocols. Patients received oxazepam 10 mg/po at the night before surgery, promethazine 25 mg/ po 1 hour before surgery, and clonidine 0.1 mg/po before surgery as premedication. Patients were NPO (except medication) for 8 hour before surgery.

During surgery all patients had standard continuously monitoring of ECG, pulse oximetery, invasive blood pressure monitoring, non- invasive blood pressure monitoring, central venous pressure, end tidal capnometery, central and peripheral temperature monitoring, bipectoral index, and also intermittent monitoring of arterial blood gas, Na, K, BS, CBC, ACT test for heparin loading & reversal. All patients received the same anesthetic regimen and routine CPB management. Anesthesia was induced by midazolam (0.05 mg/kg), fentanyl (5 mcg/kg), propofol 2 mg/kg and pancuronium (0.1 mg/kg), and was maintained with propofol infusion (10 mg/kg/h) and additional doses of fentanyl & pancuronium.

Just after ICU admission, cuff pressure was controlled and it has monitored every 8 hrs. The ventilator machine was set on SIMV-PSV mode (SIMV: Synchronized Intermittent Mechanical Ventilation, PSV: Pressure Support Ventilation), based on tidal volume = 8 cc/kg of ideal body weight, F (IMV) = 10/min, F (IMV + PSV) = 15/min, I/E (Inspiratory/Expiratory) = 1/2, PEEP (Positive End-Expiratory Pressure) = 5 cmH_2_O, PSV = 5 cmH_2_O above PEEP (total inspiratory pressure support = 10 cmH_2_O), pressure support = 10 cmH_2_O, oxygen flow = 60 lit/min and FiO_2_ =50% at first, then adjusted based on PaO_2_. Subsequently ventilation was adjusted based on ABG, and other laboratory and clinical parameters. Furthermore, RSBI (rapid shallow breathing index), which is the proportion of respiratory rate/ tidal volume, has been considered as a criteria for the extubation; if this proportion was >105, patients could not stand the extubation. The recruitment maneuver was done for all of patients before extubation.

*Specimen collection:* A 5 ml sample of blood was obtained from each patient after receiving HS or NS, just before operation and 24 and 48 hrs post-operatively, and anonymously referred to local laboratory to be centrifuged and the serum stored at −70°^C^. Plasma IL-6 & IL-10 levels were measured. We also monitored heart rate, systolic and diastolic blood pressure, central venous pressure, arterial pH, PaO_2_, FIO_2_, blood sugar, Na, K, Mg, hemoglobin, white blood cell, hematocrit and platelet for each patient before and after surgery.

*Marker measurements:* Enzyme-Linked Immunosorbent Assay (ELISA) technique was used to measure IL-6 (BMS213/2CE) and IL-10 (BMS215/2CE) (eBioscience®, Austria) samples plasma level according to manufacturer’s instructions.

*Statistical Analysis:* Data were analyzed using the statistical software SPSS version 15.0 for windows (SPSS Inc, Chicago, IL). Continuous variables are presented as mean ± standard deviation (SD) or mean ± standard error of mean (SEM), while categorical variables are summarized by absolute frequencies and percentages. Continuous variables were compared using the Student's *t*-test or nonparametric Mann–Whitney *U* test, whenever the data did not appear to have normal distributions; and categorical variables were compared using Chi-Square test, as required. Repeated measure ANOVA was used to evaluate inter-group differences and intra-group changes. All p values <0.05 were considered statistically significant.

## Result

An equal number of patients received NS or HS. There were no differences in demographic data (Table 1). Detailed clinical information of HS and control group has been summarized in Table 2.

**Table 1:** Demographic Data

**Variables**		**Hypertonic Saline (5%)**	**Normal Saline (0.9%)**	**P-value**
Number of patients		20	20	
Gender (male/female)	n(%)	14(70)/6(30)	11(55)/9(45)	0.270
Age (years)	*	62.15 ± 7.51	61.35 ± 8.91	0.760
Weight (Kg)	*	73.25 ± 11.29	69.80 ± 12.77	0.371
Height (Cm)	*	166 ± 9.20	163 ± 8.58	0.301
Diabetes Mellitus	n(%)	6(30)	10(50)	0.197
Unstable angina	n(%)	3(15)	3(15)	0.669
Opium user	n(%)	3(15)	2(10)	0.999

* Values are presented as Mean ± SD.

**Table 2:** Clinical Data

**Variables**		**Hypertonic saline (5 %)**	**Normal saline (0.9 %)**	**P-value**
PRE-OPERATIVE DATA				
Hemoglobin (Hgb) (g/dl)	*	13.93 ± 2.02	13.44 ± 1.67	0.410
Hematocrit (Hct) (%)	*	40.97 ± 4.22	40.50 ± 4.22	0.727
Platelet (PLT) (n/mcL)	*	209616.67 ± 65771	197915 ± 59753	0.57
White Blood Cell (WBC) (n/mcL)	*	7143 ± 1831.92	7200 ± 2045.04	0.933
INTRA-OPERATIVE DATA				
Pump Time (min)	*	56.70 ± 18.16	61.22 ± 13.61	0.397
Cross Clamp Time (min)	*	30.36 ± 6.79	31.82 ± 7.10	0.629
O_2_ Pressure (mmHg)	*	221.70 ± 123.6	234 ± 107.67	0.744
Serum Bicarbonate (mmole/L)	*	22.05 ± 4.02	22.75 ± 2.15	0.515
POST-OPERATIVE DATA				
Inotrope use during first 24 h postoperative	n(%)	4(20)	2(10)	0.366
Systolic blood pressure (SBP) <85 mmHg	n (%)	4(20)	5(25)	0.999
Diastolic blood pressure (DBP) <70 mmHg	n (%)	12(60)	14(70)	0.507
SBP just after anesthesia (mmHg)	*	108.84 ± 18.77	110 ± 10.64	0.813
DBP just after anesthesia (mmHg)	*	63 ± 14.24	66.90 ± 10.16	0.329
Heart Rate (1 h after surgery) (beat/min)	*	85.55 ± 14.460	88.10 ± 12.039	0.548
Heart Rate (3 h after surgery) (beat/min)	*	84.90 ± 13.114	88.10 ± 10.336	0.397
Central Vein Pressure (1 h after surgery) (cmH_2_O)	*	11.25 ± 4.518	9.45 ± 4.186	0.199
Central Vein Pressure (3 h after surgery) (cmH_2_O)	*	11.25 ± 4.50	9.88 ± 4.232	0.345
Intubation Duration (hr)	*	14.08 ± 4.17	13.98 ± 3.80	0.943
Serum Sodium (Na) (mg/dl)	*	141.45 ± 4.85	142.80 ± 3.80	0.334
Serum Potassium (K) (mg/dl)	*	4.37 ± 0.50	4.28 ± 0.44	0.531
Serum Magnesium (Mg) (mg/dl)	*	2.08 ± 0.14	2.08 ± 0.17	0.959

*Values are presented as Mean ± SD.

Of the 40 patients included, 4 in HS group versus 2 in NS group had received inotropic drug within the first 24 hrs after surgery, however the different was not statistically significant (0.336) (Table 2). Plasma concentration of sodium was nearly similar in two groups which indicate that hypernatremia was not happened as a side effect of HS use. Also serum concentration of other electrolytes (potassium & magnesium) were not different between two groups (Table 2).

**Figure 1:**
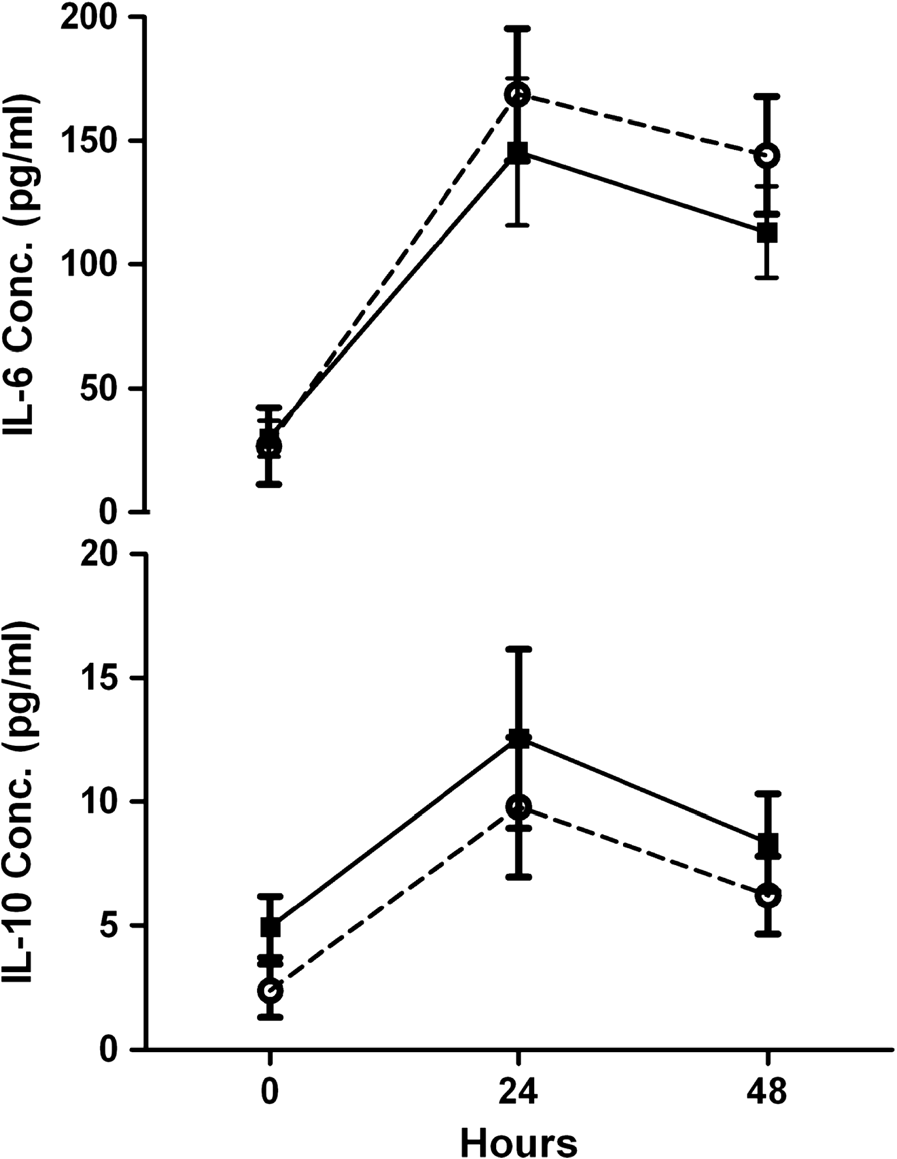
**Changes in plasma concentration of IL-6 and IL-10 for hypertonic saline vs. normal saline by hours. **Solid squares, hypertonic saline; open circles, normal saline.

The plasma levels of pro-inflammatory (IL-6) and anti-inflammatory (IL-10) cytokines were significantly changed during 48 hrs post-operatively in two groups (p < 0.001 & p < 0.005 respectively) (Figure 1). The mean of IL-6 levels at 24 hrs and 48 hrs post-operatively in HS group were lower than NS group, but this differences were not statistically significant (p = 0.437). The mean of IL-10 levels pre-operatively and at 24 hrs and 48 hrs post-operatively in HS group were higher than NS group, but the mean levels between two groups were not statistically significant (p = 0.276).

IL-6 levels rose significantly during 24 hrs in both groups but its changes were more considerable in NS group in comparison to HS group, while the mean level was 145.53 pg/ml 24 hrs post-operatively in HS group versus 174.41 pg/ml in NS group. We had a decline in IL-6 levels between 24–48 h after surgery in two groups; the mean level after 48 h was 113.13 pg/ml for HS group and 146.97 pg/ml for NS group (Table 3, Figure 1). During the first 24 h after surgery IL-10 levels in two groups rose from baseline and its level in HS group (mean value: 12.55) was higher than NS group (mean value: 10.04). IL-10 levels manifested a decline 48 h after operation and mean IL-10 level was 8.35 in HS group and 6.24 in NS group. (Table 3, Figure 1).

**Table 3:** Cytokines concentration changes

**Variables**	**Hypertonic Saline 5%**	**Normal Saline 0.9%**	**p-value**
IL-6 levels right before surgery	29.69 ± 7.26811	27.73 ± 14.32458	0.85
IL-6 levels 24 h postoperatively	145.53 ± 29.80616	174.41 ± 26.89984	0.57
IL-6 levels 48 h postoperatively	113.13 ± 18.64981	146.97 ± 24.28702	0.31
IL-10 levels right before surgery	4.95 ± 1.24330	2.49 ± 1.10323	0.12
IL-10 levels 24 h postoperatively	12.55 ± 3.63537	10.04 ± 2.89789	0.55
IL-10 levels 48 h postoperatively	8.35 ± 1.97194	6.24 ± 1.62633	0.40

*Values are presented as Mean ± SEM. Values are in pg/ml.

## Discussion

Patients undergoing CABG surgery with CPB, experience many aggressive factors, including operative trauma, cardioplegia, ischemia-reperfusion injury and the contact of blood with bioactive surfaces that potentially related to a whole body inflammatory response [8]. Although this inflammatory reaction to CPB often remains at subclinical levels, it can also lead to important clinical implications. In 1999 the Society of Thoracic Surgeons National Database, reported that 20% of “low-risk” patients developed post-operative complications [9]. Another work in these patients had shown the incidence of multiple organ dysfunction syndrome (MODS) following CPB was 11%, with a mortality rate of 41% [6]. Various studies have investigated different methods to reduce this vigorous inflammatory response following cardiac surgery with CPB; include perioperative administration of corticosteroids [10], aprotinin [11], statins [12], pentoxifylline [13], milrinone [14], ketamine [15], and bovine intestinal alkaline phosphatase [16].

A review article by Pasnik J [17] indicates that mechanism of inflammatory response to CPB is due to neutrophil activation, degranulation and endothelial dysfunction.

Ischemia-reperfusion injury that often occur following aortic declamping, cause a significant elevation in plasma cytokine levels during and after cardiac surgery under CPB. Consequences of reperfusion injury in major organs such as heart, lung, and central nervous system, include myocardial stunning and alteration in beta-receptor function (that related to transient and chronic myocardial ischemia respectively) [18]; acute respiratory distress syndrome (ARDS) and prolonged mechanical ventilation [19]; delirium, encephalopathy, stroke and cognitive brain dysfunction [20]; might infringe the clinical benefit of interventions employing extracorporeal circulation and clamping of the aorta.

HS solutions have been examined in numerous studies as plasma volume expanders for resuscitation in various hypovolemic situations but there is little attention to their immunomodulatory effects. Basic science studies have demonstrated that HS has marked effects on the immune system. More investigations have confirmed that HS blunted neutrophil (PMNs) activation and diminished the monocytes production profile in patients with traumatic hemorrhagic shock [1]. HS solution also reduced pro-inflammatory tumor necrosis factor (TNF)-alpha production significantly, while increasing anti-inflammatory IL-10, thus altering the balance between pro-inflammatory and anti-inflammatory cytokines [4,5]. Coelho and collageous [21], demonstrated that expression of cyclooxygenase (COX)-2 and inducible nitric oxide synthase (iNOS) was markedly increased in the pancreas of the acute pancreatitis patients and was reduced by treatment with HS. HS also reduced the levels of TNF-alpha and IL-6 but not of IL-10 in the pancreatic tissue. In another study, Coimbra and collageous [22] demonstrated that HS reduce traumatic shock induced T-cell dysfunction.

IL-6 is produced by monocytes, lymphocytes, and endothelial cells. IL-6 induces the adhesive neutrophil-cardiac myocyte interaction and myocardial damage following CPB surgery. Highest plasma level of IL-6 significantly correlated with the duration of SIRS [23] and also outcome in other setting of SIRS positive patients like [24-27]. Results of this study indicated anti-inflammatory effect of pre-operative administration of HS in patients undergoing CABG (Figure 1). IL-6 plasma levels were lower (but not statistically significant) in HS group during 48 h after surgery. This effect may be justified by HS osmotic effect. Osmotic effects of HS solution resulted in fluid shift from intracellular to the interstitial and intravascular space [1].

In the state of ischemia (duration of aortic cross clamp) endothelial surface layer thickness and glycocalyx structure have impaired, and dimension of glycocalyx degradation was proportional to the duration of ischemia [28], therefore endothelial cell membrane ion exchange has disturbed and ATP loss has occurred [1]. Destruction of the glycocalyx could be a trigger for increased trans-endothelial permeability leading to the development of tissue edema. Pre-operative use of HS induces normalization of endothelial cell volume and edema that increase capillary diameter and reduce resistance to flow. Therefore plasma viscosity is reduced as a result of the plasma volume expansion [1]. Hypertonicity has a direct relaxant effect on vascular smooth muscle with resultant arteriolar vasodilatation [1]. Overally, these physiological effects result in increased capillary blood flow and this improvement observed in all areas of microcirculation by HS [1].

After declamping aorta and reestablishing the microvascular perfusion, the ischemic process has been stopped by supplying the oxygen and nutrients, but at the same time a cascade of events has similar characteristics to inflammatory response is rapidly initiated. This inflammatory-like response to ischemia-reperfusion, mediated largely by neutrophils [29]. Stimulated polymorphonuclears (PMNs) via the expression of adhesion molecules leading to interaction between endothelial cells therefore play a critical role in extending the tissue damage [30].

IL-10 is a prototypical endogenous anti-inflammatory and immunosuppressive cytokine [31], which inhibits monocyte/macrophage activation and down-regulates the biosynthesis of TNF-α and IL-1β, while preventing their biologic actions via up-regulation of IL-1ra [32]. IL-10 also decreases leukocyte adhesion and its recruitment to sites of inflammation [31]. Together these mediators serve to limit the potentially injurious effects of excessive inflammatory reactions [32]. Several recent investigations have shown that early therapeutic administration of IL-10 is effective in preventing the initial surge in TNF-α observed after traumatic hemorrhagic shock [33], and also in reducing the systemic inflammatory response and lethality in murine models of sepsis and reperfusion injury [34].

Higher levels of IL-10 in HS group of our study may indicate its ability to evoke host anti-inflammatory cytokines to reduce reperfusion damage.

HS solutions have benefits on cardiac outputs, because of the increasing preload (venous return and volume expanding effect) and decreasing afterload (vasodilation and reduction in pulmonary and systemic vascular resistance) [1,35]. Whether cardiac output improving, because of the direct effect of HS on increasing cardiac contractility and having inotropic effect or due to preload effect must be further investigated [1,35]. In our study we have found that patients receiving HS required inotropic support more frequently comparing to normal saline group (4 vs. 2 p = 0.366) in the first 24 h after surgery, that may refute cardiac benefits of HS, but not statistically significant. We can explain this phenomenon, that myocardial stunning or altering in beta-receptor function as a result of ischemia-reperfusion injury occurred in some of them. Moreover patients receiving HS had lower systolic and diastolic pressure in comparison with NS receiving group which can due to possible transient hypotensive effect after administration of hypertonic saline, which was noted by Boldt J and collageous's [36]. We can suggest designing another study using HS during CPB, because in this period dropping of blood pressure is more considerable. Looking at the positive clinical trend of our patient’s treatment groups challenged with HS, higher dose of HS could be considered in a larger sample size study following CABG to reach improved statistical and clinical results.

Limited number of eligible candidate for intervention with hypertonic saline before CABG, uni-center nature of the study, high cost of cytokine evaluation and ethical concern regarding the safety of hyperoncotic fluids on subject with venerable hemodynamic profiles were mean parts of our study’s limitations.

## Conclusion

Pre-treatment with small volume hypertonic saline may have beneficial effects on inflammatory response following CABG operation.

## Competing interests

The authors declare that they have no competing interests.

## Authors contribution

MM was responsible for data gathering and manuscript preparation. FY was responsible for study design, patient selection, data gathering, manuscript preparation, MA in-charged for statistical analysis, cytokine bio-assays. HH was responsible for manuscript preparation. KB was responsible for patients’ selection. MAB was responsible for sample preparation and Laboratory tests. AJ was responsible for statistical analysis. AA was in-charged for study design and proposal preparation. SM was involved in study design. MB was involved in data gathering. MM was mean main scientific manager of the study, and was involved in design of the study and proposal preparation, responsible for mean main idea, and discussion for finding of study. All authors read and approved the final manuscript.
